# Supplementary material for: The Impact of Voluntary Policies on Parents’ Ability to Select Healthy Foods in Supermarkets: A Qualitative Study of Australian Parental Views
Source: Int J Environ Res Public Health. 2019 Sep 12;16(18):3377. doi: 10.3390/ijerph16183377 (PMC6765896; doi:10.3390/ijerph16183377)
Supplement: Supplementary file 1 [file ijerph-16-03377-s001.zip › Supp Table 1.pdf]

Supplementary Table 1: Consolidated criteria for reporting qualitative studies (COREQ): 32-item checklist[#]

No	Item	Guide questions/description	Response
**Domain 1: Research team and reflexivity**			
Personal Characteristics			
1.	Facilitator	Which author/s conducted the interview or focus group?	Employed facilitator
2.	Credentials	What were the researcher's credentials?	Experienced qualitative researcher
3.	Occupation	What was their occupation at the time of the study?	Market researcher
4.	Gender	Was the researcher male or female?	Female
5.	Experience and training	What experience or training did the researcher have?	20 years' experience
Relationship with participants			
6.	Relationship established	Was a relationship established prior to study commencement?	Not relevant
7.	Participant knowledge of the interviewer	What did the participants know about the researcher?	Not relevant
8.	Interviewer characteristics	What characteristics were reported about the facilitator?	None
**Domain 2: Study design**			
Theoretical framework			
9.	Methodological orientation and theory	What methodological orientation was stated to underpin the study?	Inductive content analysis
Participant selection			
10.	Sampling	How were participants selected?	Purposive
11.	Method of approach	How were participants approached?	Telephone
12.	Sample size	How many participants were in the study?	37
13.	Non-participation	How many people refused to participate or drop out?	None
Setting			
14.	Setting of data collection	Where was the data collected?	Market research facilities
15.	Presence of non-participants	Was anyone else present apart from the participants and researchers?	No
16.	Description of sample	What are the important characteristics of the sample?	See Table 2
Data collection			
17.	Interview guide	Were questions, prompts, guides provided by the authors? Was it pilot tested?	See Table 3
18.	Repeat interviews	Were repeat interviews carried out? If so, how many?	Not relevant
19.	Audio/visual recording	Did the research use audio or visual recording to collect the data?	Yes
20.	Field notes	Were field notes made during and/or after the interview or focus group?	Not relevant

No	Item	Guide questions/description	Response
21.	Duration	What was the duration of the interviews or focus group?	90 minutes
22.	Data saturation	Was data saturation discussed?	Yes
23.	Transcripts returned	Were transcripts returned to participants for comment and/or correction?	No
**Domain 3: Analysis and findings**			
Data analysis			
24.	Number of data coders	How many data coders coded the data?	2
25.	Description of the coding tree	Did authors provide a description of the coding tree?	No
26.	Derivation of themes	Were themes identified in advance or derived from the data?	Inductively derived
27.	Software	What software, if applicable, was used to manage the data?	NVivo11
28.	Participant checking	Did participants provide feedback on the findings?	No
Reporting			
29.	Quotations presented	Were participant quotations presented to illustrate the themes/findings? Was each quotation identified?	Yes
30.	Data and findings consistent	Was there consistency between data presented and the findings?	Yes
31.	Clarity of major themes	Were major themes clearly presented in the findings?	7 major themes presented
32.	Clarity of minor themes	Is there a description of diverse cases or discussion of minor themes?	Minor themes are not presented separately

[#] Tong, A., Sainsbury, P., & Craig, J. (2007). Consolidated criteria for reporting qualitative research (COREQ): a 32-item checklist for interviews and focus groups. *International Journal for Quality in Health Care, 19*(6), 349-357. doi:10.1093/intqhc/mzm042
