# Supplementary material for: The Impact of Voluntary Policies on Parents’ Ability to Select Healthy Foods in Supermarkets: A Qualitative Study of Australian Parental Views
Source: Int J Environ Res Public Health. 2019 Sep 12;16(18):3377. doi: 10.3390/ijerph16183377 (PMC6765896; doi:10.3390/ijerph16183377)
Supplement: Supplementary file 1 [file ijerph-16-03377-s001.zip › Supp Figure 1.pdf]

Supplementary Figure 1: Food items used as stimuli for focus groups

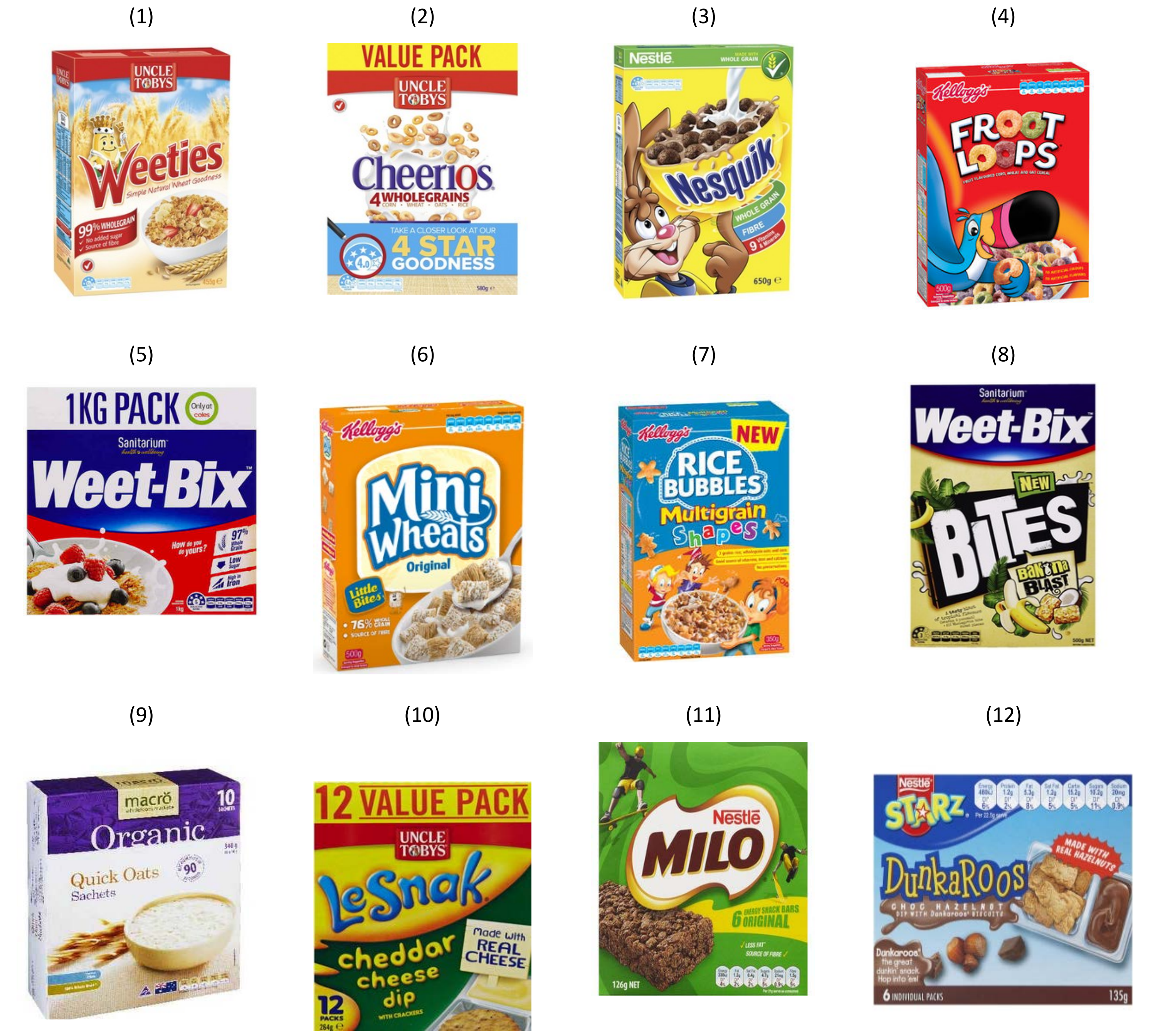

(13)

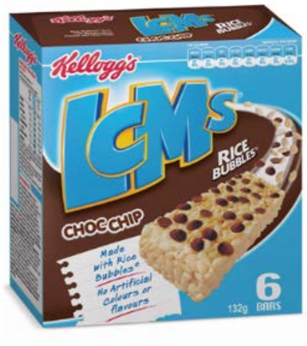

(14)

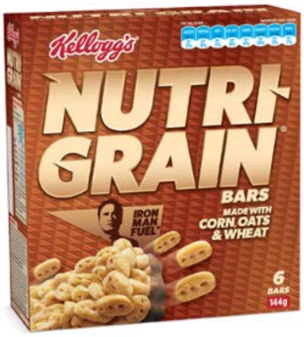

(15)

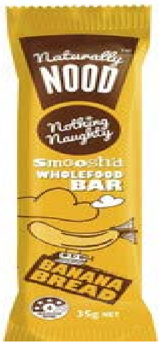

(16)

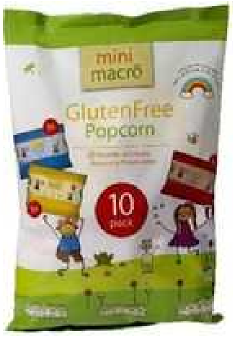

(17)

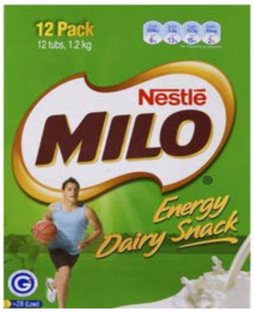

(18)

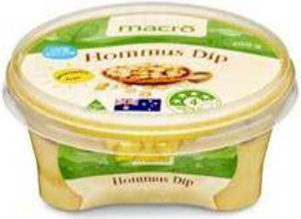

(19)

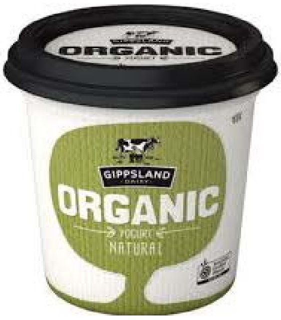

(20)

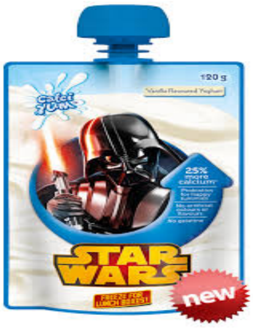

(21)

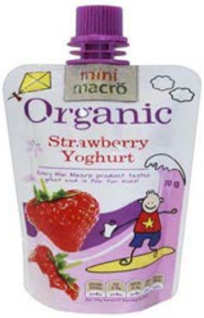

(22)

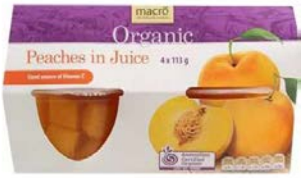

(23)

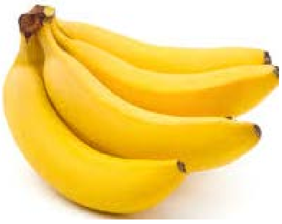

(24)

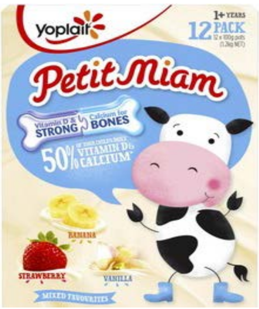

(25)

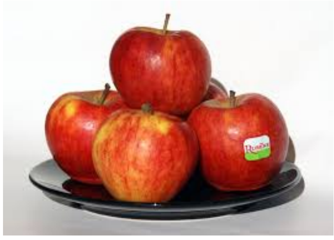

Footnote: Images were sourced from supermarket online shopping websites or food manufacturer websites.
